# Spatial Effects on the Multiplicity of *Plasmodium falciparum* Infections

**DOI:** 10.1371/journal.pone.0164054

**Published:** 2016-10-06

**Authors:** Stephan Karl, Michael T. White, George J. Milne, David Gurarie, Simon I. Hay, Alyssa E. Barry, Ingrid Felger, Ivo Mueller

**Affiliations:** 1 Population-Based Biology Division, Walter and Eliza Hall Institute of Medical Research, Parkville, Victoria, Australia; 2 Department of Medical Biology, University of Melbourne, Parkville, Victoria, Australia; 3 Vector-borne Diseases Unit, Papua New Guinea Insititute of Medical Research, Madang, Madang Province, Papua New Guinea; 4 MRC Centre for Outbreak Analysis & Modelling, Department of Infectious Disease Epidemiology, Imperial College, London, United Kingdom; 5 School of Computer Science and Software Engineering, The University of Western Australia, Perth, WA, Australia; 6 Department of Mathematics, Applied Mathematics and Statistics, Case Western Reserve University, Cleveland, Ohio, United States of America; 7 Institute for Health Metrics and Evaluation, Seattle, Washington, United States of America; 8 Fogarty International Center, National Institutes of Health, Bethesda, Maryland, United States of America; 9 Department of Medical Parasitology and Infection Biology Swiss Tropical and Public Health Institute, Basel, Switzerland; 10 University of Basel, Basel, Switzerland; 11 Malaria: Parasites and Hosts Unit, Department of Parasites & Insect Vectors, Institut Pasteur, Paris, France; Universidade de Sao Paulo Instituto de Ciencias Biomedicas, BRAZIL

## Abstract

As malaria is being pushed back on many frontiers and global case numbers are declining, accurate measurement and prediction of transmission becomes increasingly difficult. Low transmission settings are characterised by high levels of spatial heterogeneity, which stands in stark contrast to the widely used assumption of spatially homogeneous transmission used in mathematical transmission models for malaria. In the present study an individual-based mathematical malaria transmission model that incorporates multiple parasite clones, variable human exposure and duration of infection, limited mosquito flight distance and most importantly geographically heterogeneous human and mosquito population densities was used to illustrate the differences between homogeneous and heterogeneous transmission assumptions when aiming to predict surrogate indicators of transmission intensity such as population parasite prevalence or multiplicity of infection (MOI). In traditionally highly malaria endemic regions where most of the population harbours malaria parasites, humans are often infected with multiple parasite clones. However, studies have shown also in areas with low overall parasite prevalence, infection with multiple parasite clones is a common occurrence. Mathematical models assuming homogeneous transmission between humans and mosquitoes cannot explain these observations. Heterogeneity of transmission can arise from many factors including acquired immunity, body size and occupational exposure. In this study, we show that spatial heterogeneity has a profound effect on predictions of MOI and parasite prevalence. We illustrate, that models assuming homogeneous transmission underestimate average MOI in low transmission settings when compared to field data and that spatially heterogeneous models predict stable transmission at much lower overall parasite prevalence. Therefore it is very important that models used to guide malaria surveillance and control strategies in low transmission and elimination settings take into account the spatial features of the specific target area, including human and mosquito vector distribution.

## Introduction

Declining malaria transmission is associated with the formation of ‘hotspots’, which are geographical regions of limited extent, where infections cluster and transmission may remain persistent. [1] Such hotspots may not be easily identifiable by routine surveillance as much of the transmission within a hotspot is likely to occur between asymptomatic individuals. [2]

The primary reason for the occurrence of transmission hotspots is that malaria transmission is not homogeneous and humans can be exposed to substantially different levels of mosquito biting on the micro-scale (i.e., on a scale of a few meters, between neighbouring households or even within the same household). Most likely both vector population related factors such as productivity of breeding sites and distance of human dwellings from these sites as well as human related factors crucially affect micro-scale variation in transmission intensity and human exposure. [3] Although Anopheles mosquitoes are known to be able to fly considerable distances (several km) [4, 5], they will prefer available hosts close to their breeding sites. [6, 7] A wide variety of human factors such as body size, bed net usage, time spent outdoors, quality of housing and even beer-consumption has previously been shown to affect human susceptibility to mosquito bites. [8–11]

Another important factor contributing to the heterogeneity of transmission is the very variable and difficult-to-quantify duration of infection in humans. [12] Some aspects influencing duration of infection, such as acquired immunity and super-infection with new clones are directly related to exposure, others, such as treatment-seeking behaviour are less dependent on exposure. [13, 14] For example, people living a considerable distance from a health centre will seek malaria treatment less frequently leading to longer average durations of infection. [15] From the interplay of these different aspects complex patterns may arise: for example, infants, who still carry maternal antibodies (e.g., < 6 months of age) and very small children who stay mostly indoors and are closely monitored by their parents (e.g., < 2–3 yrs. of age) are often shown to harbour infections less frequently. [16] If they do get infected, they will receive treatment more often, especially since they will develop symptomatic infections more frequently leading to comparatively short durations of infection and thus a smaller overall contribution to onward transmission. [17–19] In contrast, older children and young adults (e.g., 5–15 yrs. of age) who spend more time outdoors are usually found to carry infections more frequently. However, due to a higher degree of immunity these infections are more often asymptomatic. [18, 20, 21]

Incorporation of heterogeneity in transmission represents a challenge for mathematical transmission models of vector borne diseases. Previous mathematical modelling studies have shown that heterogeneity of exposure, for example, based on the ‘80/20-rule’ (i.e., a scenario where 20% of the human population receives 80% of mosquito bites) may contribute considerably to sustaining transmission by leading to increased estimates of the basic reproduction number, R_0_. [3] However, few studies have modelled the spatial aspects, specifically the geographical distribution of humans and mosquitoes on the micro-scale (i.e., with a spatial resolution of a few meters), an important factor underlying this heterogeneity in transmission intensity. [22–26]

Infections with multiple parasite clones are common in high transmission settings and it has been hypothesized that the observed multiplicity of infection (number of clones per person, MOI) may be a good indicator of the level of transmission in a population. MOI is calculated by counting the number of genetically distinct clones detected in infected individuals. MOI must therefore take values of ≥1. Many studies report ‘mean MOI’, although it is unlikely that MOI follows a simple Poisson distribution in a population where transmission is heterogeneous. [3] Heterogeneity also impacts the relationship between MOI and parasite prevalence (another surrogate measure of transmission). Individuals residing within local transmission hotspots may be subject to much higher rates of infectious bites, therefore maintaining higher MOI levels even at very low (i.e., < 1%) overall parasite prevalence in the larger population. [27] Previous studies have shown that at low parasite prevalence average MOI is often higher than what can be explained by the random mixing of parasite clones, humans and mosquitoes in homogeneous transmission models (for a list of relevant studies describing MOI based on merozoite surface protein 2 (*msp2*) genotyping, see supporting information S1 Table). [28]

Using a spatial mathematical transmission model that allows for the transmission of multiple parasite clones, we therefore investigated whether spatially heterogeneous human and mosquito populations better explain the observed mean MOI *vs*. mean parasite prevalence relationship. [3, 7, 16, 23, 29] Based on the underlying spatial distributions of human and mosquito populations, limited mixing between these populations will arise from i) the constrained radius of mosquito flight and ii) the distance dependent movement of human individuals. [29, 30] Coupled with other factors (some mentioned above) that determine human exposure, this will inherently result in a small proportion of the population receiving most mosquito bites. As opposed to imposing an ‘80/20-rule’, these spatial features may better capture heterogeneous transmission and result in more versatile and realistic models.

Most currently used mathematical models for malaria and other vector borne infectious diseases do not explicitly consider spatial features of transmission but assume a homogeneous mixture of human individuals and mosquitoes. [23, 31–37] This may be appropriate for many aspects of transmission especially for scenarios of high transmission. However, in the modelling of very low transmission scenarios where the mosquito distribution is more heterogeneous, the actual spatial distribution of human and mosquito populations may need to be taken into account in order to provide reliable estimates. Such low transmission scenarios will be dominant in the future if malaria incidence continues to decline as expected.

The model presented here was calibrated using the spatial village structure, human age distribution and household composition (number of inhabitants and family structure) as present on the North Coast of Papua New Guinea (PNG) to ensure realistic simulations. [38] Our recent studies in this area also provided detailed data on the number circulating *msp2* alleles, overall malaria prevalence and MOI. [18, 27, 39–41]

## Materials and Methods

**Fig 1:**
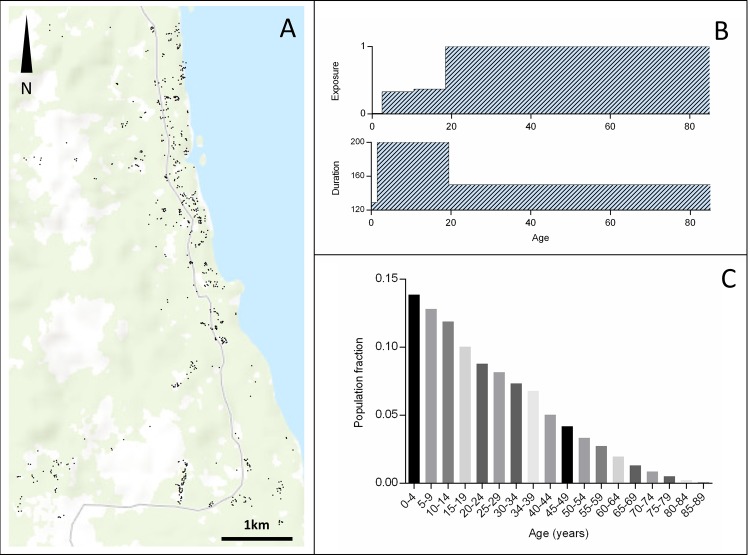
Modelling Area and Characteristics of the Modelled Human Population. Panel A: Geographical distribution of households (black dots = households); Panel B: Age dependent exposure [9] and duration of infection [12, 43]; Panel C: Rural population age distribution in Madang Province, PNG based on the current census data. [38]

We used an individual based, spatially explicit model with individual humans and mosquitoes. [42] The human population in the model (n = 3461) was based on the age distribution and household structure of rural Madang Province, PNG. [38] We used a set of geo-referenced households (n = 663) in an area of approximately 35 km^2^ (Fig 1).

*Anopheles farauti s*.*l*. is the main vector in coastal Madang. [44] It has been shown that *A*. *farauti* generally fly less than 50 m after blood feeding and have a memorized home range, whereas the other, less abundant species in the area (e.g., *A*. *punctulatus* and *A*. *koliensis*) may disperse further. [45, 46]. Another circumstance that will limit mosquito dispersal in coastal PNG is the relative absence of non-human hosts as there are only few domestic animals (pigs, few cattle). These animals are usually kept next to, or under the houses at night in PNG. [46, 47] As in previous modelling approaches, we assumed that mosquitoes are confined to the households of a specific geographical area and predominantly feed on humans residing in households within this area. [48] Within household exposure and duration of infection are dependent on human age (Fig 1). [9] It is also possible for mosquitoes to bite humans outside their area, however this probability decreases with the square of the distance between individual human and mosquito locations (households).

The probability that a specific mosquito (M_*j*_) bites a specific human (H_*i*_) is:

$$P(H_{i}/M_{j})=we_{a,i}/{\left(1+qd_{ij}\right)}^{2}$$ (1)

In Eq (1), *d* is distance between a human and a mosquito (if human and mosquito are within the same household then *d* = 0), *e*_*a*_ is a weight of human exposure related to age (*a*) based on [9] with a value ≤ 1, *w* is the biting rate and *q* is the scaling factor that determines the extent of human/mosquito mixing. Note that *q* does not represent the maximum distance that mosquitoes are physically able to fly but is related to the range of host seeking in the presence of an ample host reservoir in close proximity of the mosquito’s present location. We assume that for transmission from human to mosquito, clones are transmitted independently from each other with the same probability of transmission per bite *a*. [49] For transmission from mosquito to human it is assumed that all clones present in the salivary glands of the mosquito are transmitted simultaneously with a success rate *b*.

Multi-clone infections are acquired either by humans being bitten by one mosquito infected with multiple clones or infected humans being bitten again (on the same day, or a subsequent day), by different infected mosquitoes. If a human is superinfected with the same clone, duration of infection can be extended (a new independent infection process for the same clone is started parallel to the existing one). If a human is infected with multiple strains, these are cleared independently. Similarly, in theory, mosquitoes can bite other infected humans and acquire more infections however, due to the limited mosquito life-span, this occurs very infrequently.

**Fig 2:**
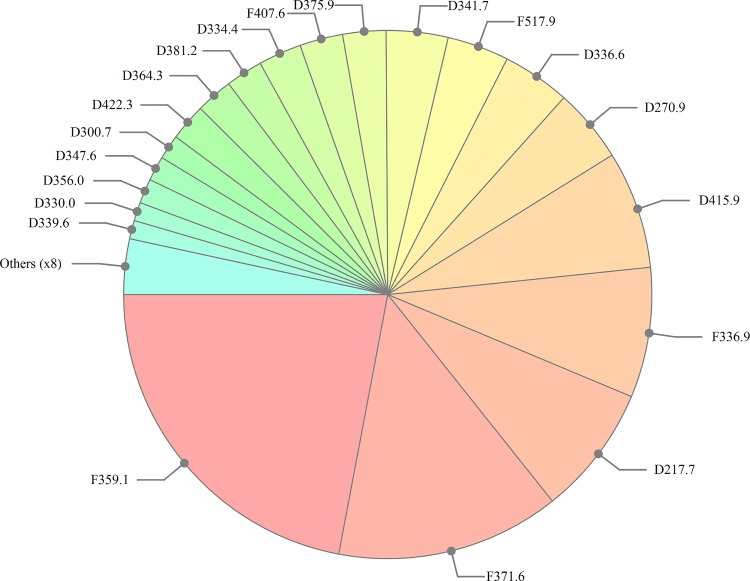
*Plasmodium falciparum* Clone Distribution Used in the Present Study. This distribution resembles that in lowland coastal PNG based on *msp2* genotyping data collected in a recent cohort study in the same region of PNG (data unpublished).

On each simulation day, travellers can acquire single or multi-clone infections similar to humans that are present in the modelling area. The force of infection acting on travellers is assumed to be constant and independent of that within the modelling area. This causes infections to be occasionally introduced to the modelling area, and overall strain distribution to be maintained. The clone distribution used in the present study is shown in Fig 2.

Humans remain infected for a duration that is dependent on age, based on previous field observations and modelling [12, 14, 43] and are assumed to remain infectious for the entire duration of infection. Mosquitoes undergo an extrinsic incubation period of the average duration 1/*n* (12 days) and live on average *1/μ* of 10 days. [50]

**Fig 3:**
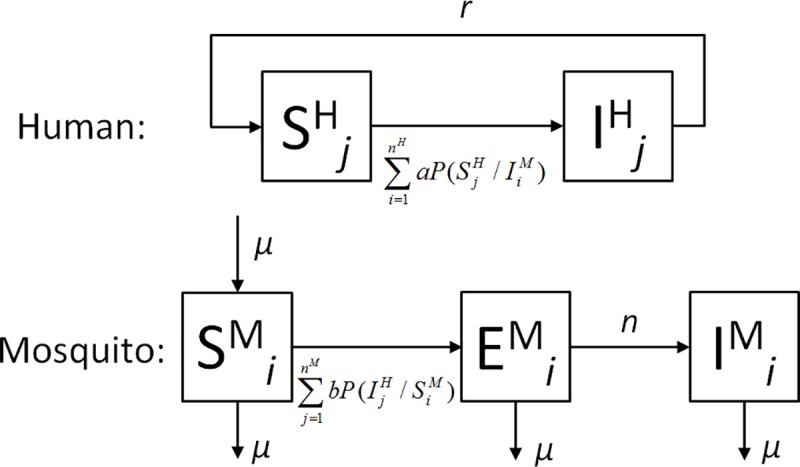
Model Used in the Present Study. Humans are either susceptible (S) or infected/infectious (I). Correspondingly, mosquitoes are either susceptible (S), within the extrinsic incubation period (E) or within the infectious period (I). Superscripts *H* and *M* refer to humans and mosquitoes, respectively. *n*^*M*^ an *n*^*H*^ refer to the total number of mosquitoes and humans, respectively. As in most previous models of malaria transmission, all durations of residence in these states are assumed to be exponentially distributed (constant transition probabilities). The indices *i* and *j* indicate that we use an individual based approach in which each human and each mosquito is represented as an individual object. *P(S*^*H*^_*j*_*/I*^*M*^_*i*_*)* and *P(I*^*H*^_*j*_*/ S*^*M*^_*i*_*)* denote the distance weighted probability (Eq 1) that a human (*j*) is bitten by a specific mosquito (*i*), whereas *a* and *b* are the probabilities of transmission given a potentially infectious bite.

The model is implemented as a simulation in which individual humans and mosquitoes are objects with properties such as geographic location and infection status (*susceptible-S*, *infected-E*, *infective*,*-I*). Transmission and progression between the infection states are stochastic processes, with fixed probabilities (as given in Table 1), and follow the standard approach (Fig 3). Mosquitoes are assumed to bite with a frequency *w*, meaning that at each day (simulation time-step), a proportion ~*w* of the mosquito population is randomly chosen (i.e., assuming an exponential distribution of biting frequency per mosquito) and assigned to bite humans (multiple bites per human are possible). The choices of the human/mosquito pairs are based on distance between humans and mosquitoes and on human exposure characteristics as specified by Eq (1) and shown in Fig 1. Only the adult, female mosquito population is modelled and ‘dead’ mosquitoes are replaced by new mosquitoes resulting in a constant mosquito population, equivalent to widely used approaches. [51]

**Table 1:** Parameters Used in the Present Study.

Parameter	Description	Value		Ref.
*Transmission probability*					
*a*	human to mosquito	0.23	[54]		
*b*	mosquito to human	0.5	[55]		
*Human/Parasite*					
*s*	number of clones^*1*^	28	unpublished		
*r*	rate of clearance of infections				
	*<2 yrs*	1/129 day^-1^	[43]		
	*2–19 yrs*	1/200 day^-1^	[43]		
	*>19 yrs*	1/150 day^-1^	[43]		
*e*_*a*_	weighted exposure dependent on age				
	*<2 yrs*	0.01	[9]		
	*2–10 yrs*	0.33	[9]		
	*10–18 yrs*	0.37	[9]		
	*>18 yrs*	1	[9]		
*Mosquito*					
*w*	mosquito biting frequency	0.21 day^-1^	[56]		
*μ*	1/mosquito life expectancy	0.1 day^-1^	[50, 56]		
*n*	1/duration of sporogony in mosquito	0.083 day^-1^	[50]		
*q*	distance scale of human/mosquito mixing^*2*^	0.03 m^-2^	arbitrary		
*Migration*					
*F*	fraction of adult humans travelling	0.1	arbitrary		
*t*	average travel time	14 days	arbitrary		
*p*_*out*_	probability of infection while traveling^3^	0.0–0.1 day^-1^	arbitrary		

^1^see Fig 2 for clone distribution

^2^with *q* = 0.03 m the probability that a mosquito seeks a host at a distance of 10 m is 25% of that at 0 m (same household) and 0.3% at 100 m

^3^*p*_*out*_ is a linear function based on the number of infections within the modelling area, $p_{out}=k\sum I$ where ∑I is the sum of all infections within the modelling area and *k* is a scaling factor to achieve 0.1/day probability at 100% infection rate (*k* = 1.07 x 10^−6^).

To represent the integration of the modelling area into a wider geographical region with a similar parasite clone distribution, we allow for infections to be introduced into the modelling area by human migration/travel. We assume, that on average a 10% fraction of the adult population (>14 years) is currently travelling, resulting in a constant population within the modelling area. Average travelling time per person is 14 days. Humans outside the modelling area can be infected with a probability dependent on transmission within the modelling area, and the infecting clone(s) will be chosen based on the overall clone distribution in the modelling area at the start of the simulation. (Fig 2). If transmission within the modelling area is high, introductions through migration occur more frequently but decrease linearly with decreasing transmission. More complex models describing human movement and migration have previously been developed yet their implementation is only reasonable if local human migration and movement data for the modelling area is available, which was not the case for PNG. [23, 52, 53] All model parameters are shown in Table 1.

### Different Transmission Environments

**Fig 4:**
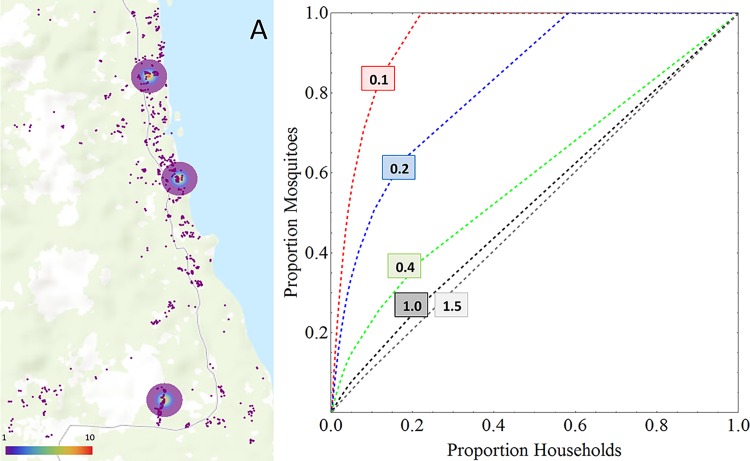
Heterogeneous Mosquito Distribution per Household in Low Transmission Settings. For the illustrative purposes of this work, we assumed 3 regions in the modelling area where mosquito density was higher than in the surrounding areas (indicated by circles in Fig 4A). The colour scale indicates the number of mosquitoes per household assumed for households located in the respective areas. Panel B shows the resulting overall proportion of mosquitoes found in the overall proportion of households. As observed in field studies, especially in low transmission settings, often a small proportion of households harbour most of the mosquitoes. A general experimental observation is that mosquito numbers become more evenly distributed as mosquito numbers increase (Supporting Information S1 Text). [57, 58] The numbers on the curves in 4B indicate the mosquito-to-human ratios resulting in the respective curves.

Following observations from the field, we assumed that in high transmission settings, mosquito density is more evenly distributed across households as compared to low transmission settings (this is further underpinned by the data analysis presented in Supporting Information S1 Text). In low transmission settings, mosquitoes were assumed to be clustered around specific breeding sites so that a smaller proportion of households sustained most of the mosquito population. In the present study 3 such mosquito population clusters were purposely generated to represent hotspots. These are represented by the circles in Fig 4A. We assumed that stable mosquito numbers exist in these clusters (e.g., related to stable bodies of water), and that mosquito numbers are decreasing with the square of the distance away from the centre of each of these hotspots (Fig 4A).

As transmission intensity increases, the areas away from these ‘hotspots’ are gradually and homogeneously populated with mosquitoes (e.g., by the formation of temporal, shallow water bodies during the rainy season) whereas within the hotspot area, mosquito numbers remain stable but not lower than the mosquito density of the surrounding landscape a characteristic e.g., observed by Ribeiro et al. in Ethiopia. [57] A Supporting Video File (S1 Video) was generated to illustrate this assumption graphically. Fig 4B shows the resulting proportion of mosquitoes *vs* proportion of households for various mosquito-to-human ratios. This resembles observations from the field (e.g., from Kenya and Ethiopia, for further analyses see Supporting Information S1 Text). [57–60]

The spatial model including heterogeneous biting based on geography, mosquito flight and age-dependent exposure was compared to two other models. Firstly, a ‘null model’, where exposure was homogeneous and parasite clones were Poisson distributed across the human population at a given prevalence, and secondly, a model with no spatial features but taking into account age-dependent within-human differences in exposure and duration of infection (shown in Fig 1B and 1C). The model was run for different mosquito-to-human ratios as shown in Fig 4A to achieve different parasite prevalence. Prevalence was then plotted against MOI. The resulting prevalence *vs* MOI pattern was compared to data compiled from a literature survey on *msp2* based observations of MOI (S1 Table). Spatial estimates for average frequency of infected bites per person per unit time (entomological inoculation rate, EIR) where derived by running the model at an equilibrium state for 30 years, mapping the average number of infectious bites per person per year and applying an inverse distance weighted interpolation algorithm to derive EIR isolines using the QGis 2.0 software.

### Model Limitations

Similarly to previous modelling studies, transmission of individual clones was regarded to be mutually independent and the present model does not account for genetic recombination in the mosquito vector. [61] Therefore, clones are not changed by passage through the mosquito. This simplistic assumption is sensible for a scenario where estimates of MOI are based on genotyping of a single marker gene such as *pfmsp2*, where new alleles will only arise through relatively rare events such as point mutations, crossing overs and /or replication errors changing the number of sequence repeats. [62] Similarly, the model does not account for clone specific acquisition of immunity in the human population. [43, 63] However, the aim of this study was to show the general effects of spatial heterogeneity on MOI and therefore these features were not considered essential.

As with previous vector borne disease models, the present model assumes a fixed spatial distribution of humans and mosquitoes in which humans and mosquitoes are predominantly associated with specific locations. [33, 48] Mixing due to human movement and mosquito flight are partially captured by the distance weighted biting given by Eq 1. [64] As with most other malaria transmission models, we do not explicitly model the mosquito life cycle (apart from the female adult stage). While epidemiological models aiming to describe vector control (e.g., [23, 65–67]) should include mosquito population dynamics, this was not within the scope of the present study.

The model is subject to the usual limitations of the widely used compartmental model systems including exponentially distributed transitions and fixed infectivity (gametocytes are not explicitly included in the model). [68, 69] Only *P*. *falciparum* transmission is considered. For *P*. *vivax*, additional considerations regarding the presence of a hypnozoite reservoir in the human population will need to be taken into account. [70] We do not explicitly account for treatment or other malaria control measures such as bed nets. Furthermore, we do not consider imperfect detectability of clones in multiple-clone infections, which is the main cause for the age *vs*. MOI relationship shown by several studies [43]. However we show that the present model can be expanded to realistically reproduce this relationship when incorporating additional data on age dependent detectability. (S1 Text)

In agent-based simulations, the number of modelled parasite clones, humans and mosquitoes is constrained by computational limitations and therefore a relatively confined modelling area and population size were chosen. [71]

## Results

### Spatial EIR estimates

**Fig 5:**
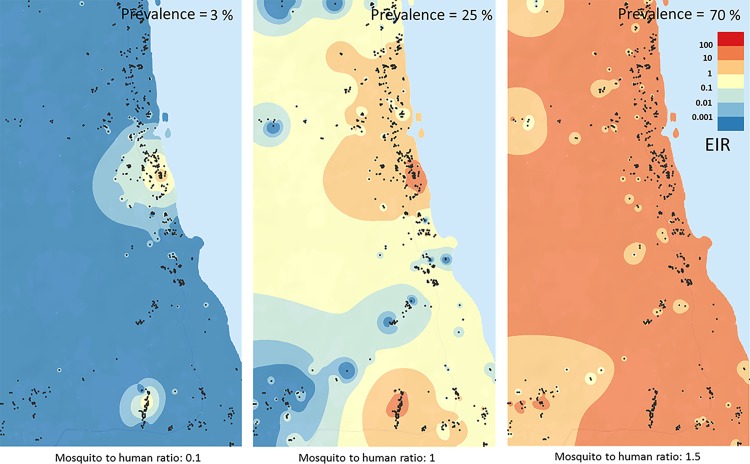
Estimated EIR Isolines for Different Transmission Settings. At low transmission (3%) transmission is highly clustered based on mosquito and household density. The heterogeneity in EIR decreases as transmission increases (25% and 70%) and the hotspots lose their clear-cut edges. The isolines were derived from infection event counts when running the model for 30 years at the respective prevalence. It can be seen especially in Panel A, that the northernmost mosquito cluster does not result in sustained transmission due to lower mosquito numbers in this cluster as explained in the text.

The clustering of mosquitoes around persistent breeding sites leads to very variable EIR estimates across the landscape in low transmission settings. This is illustrated in Fig 5 showing estimated EIR isolines for low, medium and high transmission settings [3%,25% and 70% parasite prevalence in the overall population, respectively, based on the mosquito density distribution illustrated in Fig 4 (and S1 Video)]. Fig 5A, representing the low transmission scenario, shows clearly defined hotspots. As the mosquito-to-human ratio is increased and the overall mosquito distribution becomes more homogeneous, EIR estimates also become more evenly distributed. (Fig 5B and 5C).

Fig 5 shows that, with the spatial model, even at very low average prevalence in the entire population, individuals in a confined area determined by the mosquito clusters may still be exposed to >100 infectious bites per year, whereas in most of the remaining area the probability to receive an infectious bite is near zero. The individuals within the hotspot are therefore likely to maintain higher infection prevalence and MOI levels and the MOI population average is likely to be considerably above the lower limit value of 1. Importantly, this will also facilitate the generation of genetic diversity until transmission within the hotspot itself is targeted.

Especially Panel 5A shows, that the northernmost mosquito cluster (shown in Fig 4A) does not result in sustained transmission given the same assumptions about mosquito numbers and flight as in the other clusters. The reason for this is that the centre of the cluster (with the highest mosquito density) is around a relatively isolated household (only 1 other household is within 50 m, and 5 within 100 m). Since we assume decreasing mosquito numbers away from the cluster centre, this mosquito cluster contains overall fewer mosquitoes. The other mosquito clusters contain 15 and 11 households, respectively, within a 100 m radius from the centre leading to much higher mosquito numbers and a much higher rate of exchange of infections between households.

### Average MOI with Changing Parasite Prevalence

**Fig 6:**
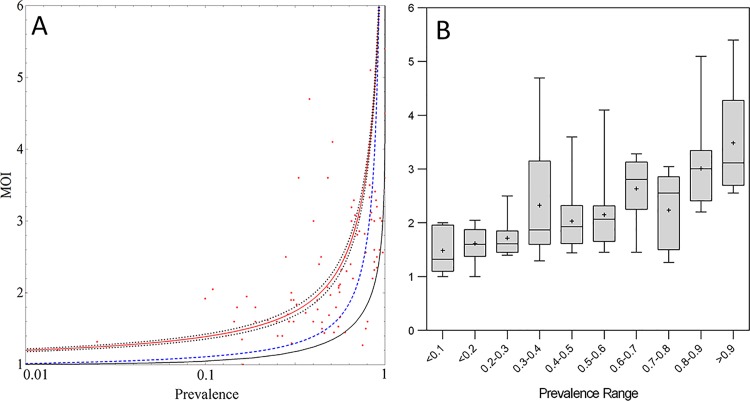
MOI *vs*. Parasite Prevalence: Spatial Model and Null Model Predictions *vs*. Field Data. Panel A shows the spatial model prediction (red, with 95% CI band), the null-model prediction (black), the model with no spatial features but age based exposure and infection duration (blue, dashed) and the field data (dots). Spatial model predictions were smoothened by appying a lowess algorithm. Panel B shows the data gathered in the literature review grouped into 10% prevalence ranges using box and whisker plots (boxes are median/interquartile ranges, whiskers are ranges).

Average MOI of the overall population in the spatially explicit simulations remained considerably above the lower limit of 1 for very low malaria prevalences (~ 1%). It should be noted that below a prevalence of around 1% (or ~35 infected individuals in the modelling area) the parasite population was not sustained, owing to the limited number of human individuals in the model. However, even if the modelling area would be much larger (e.g., containing many 10K or 100K people) it is really the size of the hotspot and the transmission intensity within the hotspot (e.g., Fig 5A) that determines prevalence. In other words, if the modelling area would be expanded but no new hotspots would be added, much lower overall prevalence would be possible, and MOI would asymptotically approach the limit value of 1. Fig 6 shows the MOI versus overall prevalence data compiled from the literature survey (S1 Table) in combination with the predictions from the spatially explicit model, the non-spatial model incorporating within-human differences and the homogeneous null model. Both, the null-model (homogeneous mixing) and also the model where only human attributes are heterogeneous but no spatial features are included, do not support field observations of MOI substantially >1 at low levels of overall parasite prevalence (e.g., at prevalence levels below 10% the null model and the non-spatial model both predict MOI levels of ~1).

### MOI Estimates for Populations Inside and Outside of Hotspots

**Fig 7:**
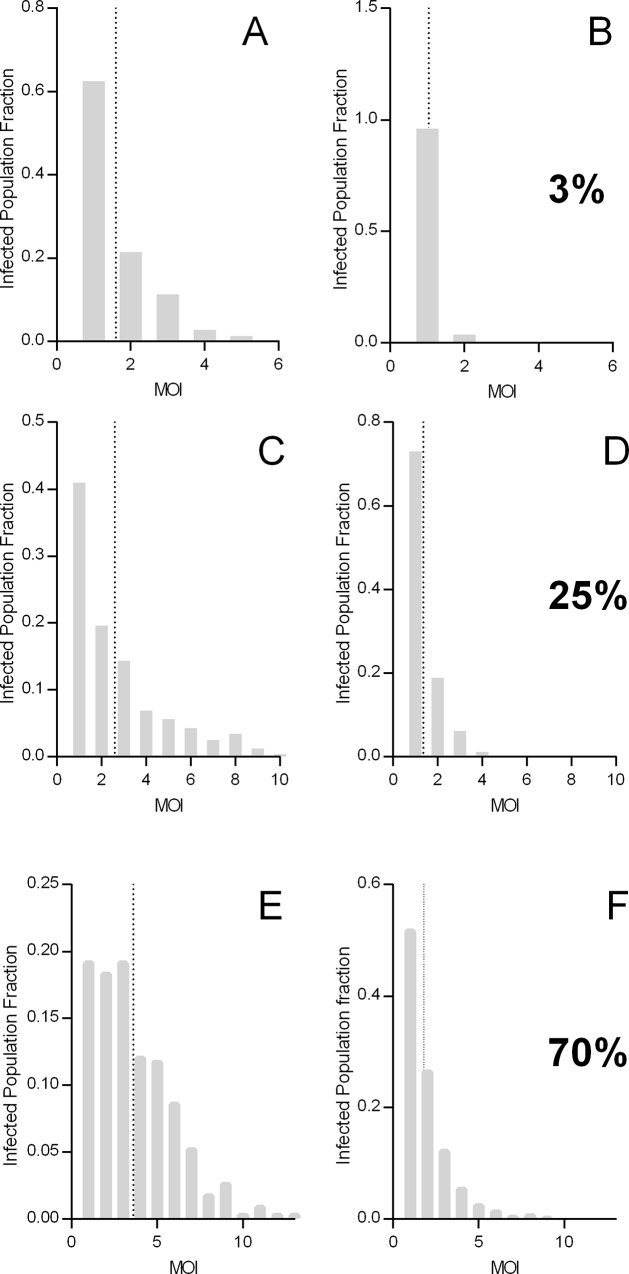
MOI in Sub-Populations Living Inside or Outside of Hotspots. The extent of the potential hotspots is given in Fig 4. The population within the hotspots exhibits higher average MOI and a higher parasite prevalence consistent with field observations. [43]

Fig 7 shows an example of predicted MOI distribution for the population living inside and outside the hotspots generated in the present model with an average mosquito-to-human ratio of 0.1, 1 and 1.5 (corresponding to the same scenarios as in Fig 5 with 3%, 25% and 70% prevalence in the spatial model). For the low transmission scenario (3% prevalence), there are stark differences in prevalence outside and inside the hotspots (0.4% outside, 13% inside and mean MOI: 1 outside and 2.1 inside). The differences decrease in the higher transmission scenarios, yet MOI is always higher in the population residing within the hotspots (2.7 *vs*. 1.4 for the 25% scenario and 3.6 *vs*. 1.9 for the 70% scenario).

### Changing Average Prevalence with Mosquito-to-Human Ratio

**Fig 8:**
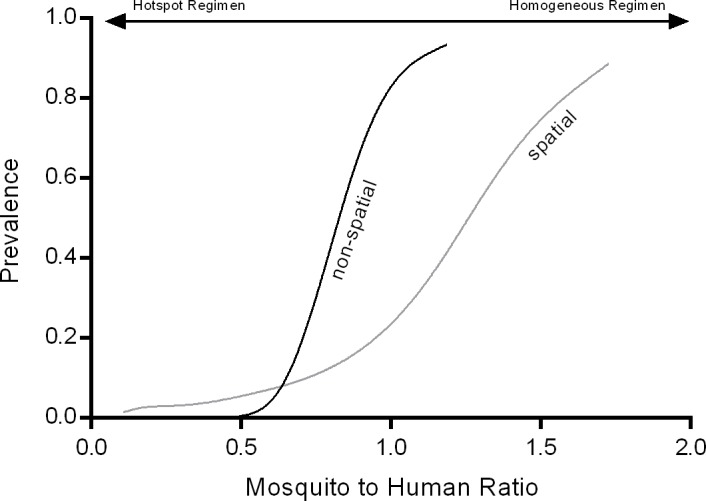
Relationship Between Mosquito-to-Human Ratio and Equilibrium Prevalence. While spatial transmission features lead to higher predicted prevalence in overall low transmission settings, these features result in a lower rise in predicted prevalence with increasing mosquito-to-human ratio (as transmission is still locally confined even at higher mosquito-to-human ratios).

Mosquito-to-human ratio is usually used to adjust the ‘transmission level’ in compartmental models. The spatial model and the null model exhibit different characteristics with regard to the relationship between mosquito-to-human ratio and the resulting equilibrium parasite prevalence as shown in Fig 8. Whereas in the non-spatial, homogeneous human population model, predicted prevalence increases within a narrow range from 0% to ~100%, the spatial model shows a slower increase but prevalence > 0% is sustained by lower mosquito-to-human ratios than in the null model. The reasons for this are i) the assumption that mosquitoes are clustered around specific households and ii) the resulting limited spatial mixing of mosquitoes and human populations.

## Discussion

The present modelling study using a spatially explicit environment with realistic geographic household distribution underlines the importance of considering spatial heterogeneity when modelling malaria transmission, especially in low transmission settings. [71] We show that the relationship between MOI and parasite prevalence is not well captured by models with homogeneous transmission as field studies have shown that even in low transmission settings, MOI levels above the limiting value of 1 are a common occurrence. [27]

From a modelling perspective, the existence of ‘hotspots’ where stable transmission occurs only within a small, and geographically focused proportion of the population can explain these observations. Within the hotspot, transmission (e.g., as measured by local or household-based EIR) can be much higher than in the surrounding areas, causing a small proportion of the population to exhibit higher MOI levels. Fig 6A shows that MOI is a nonlinear function of prevalence and especially at low transmission intensities (e.g., as indicated by overall parasite prevalence of <10%), MOI changes very little with changing overall prevalence. This implies that average MOI is unlikely to be a very sensitive measure of the overall transmission intensity in low transmission settings and that local EIR variation will need to be taken into consideration when deducing transmission intensity based on MOI. Genotyping however, may also represent an opportunity to ‘triangulate’ hotspot locations and guide focalised malaria control in very low transmission settings.

It should be noted that spatial heterogeneity is not the only type of heterogeneity that determines human exposure and infection, and thus MOI distribution in a population. Several other important factors such as age-related exposure and occupational exposure, and the related degree of acquired (clone-specific) immunity will also influence the MOI distribution in a population.

It is very likely, that Anopheles flight and host seeking behaviour significantly influences the extent and focus of transmission hotspots. Different species of Anopheles mosquitoes exhibit very different behaviour. Although it has been shown that Anopheles mosquitoes are able to fly, or be carried by wind, for >10 km, recapture rates are often very low (<1%) making it difficult to estimate the distance naturally flown by an individual mosquito, especially when an ample host reservoir is present. [72, 73] We assume that the majority of mosquitoes stay in close proximity to suitable hosts and rarely fly long distance since *Anopheles farauti s*.*l*., which is the main vector in coastal lowlands on the North Coast of PNG has been shown to exhibit this behaviour. [74–77]

All models discussed here (the spatial model, the non-spatial model incorporating inter-human heterogeneity and the homogeneous null-model) exhibit a very steep increase in MOI at prevalence rates > 80%, whereas the data collected in the literature review as part of the present study, suggests a more moderate increase. It should be noted, that all available molecular techniques to determine MOI will underestimate true MOI due to the non-detection of minority clones. Therefore, due to the fact that the present model does not account for detectability (e.g., the probability to detect a clone based on different parasite density of clones in the blood of an individual), MOI is likely to be overestimated by the models [78]. At high transmission, an increasing proportion of people are infected with minority clones for which the probability of detection is lower. [41] Therefore, it is expected that detectability reduces observed MOI at high transmission levels much more than at low transmission levels. However, data on within-host clone distribution and related detectability are very sparse. In addition, it is likely that clone specific acquired immunity and the related clone specific clinical incidence shapes regional clone abundance profiles. Studies that relate parasite genotypes with clinical incidence are required to calibrate more complex multi-clone models which include clinical incidence and treatment. [79]

Challenges for the development of models such as the one presented here, are the requirements for much more detailed parasitological (clone specific growth, clinical incidence rates), entomological (mosquito dispersion and host seeking) and human behavioural (movement and migration) data, efficient programming and supercomputer facilities to minimize run time.

However, the current model, although subject to substantial limitations, illustrates clearly that individual-based, spatial approaches are required to capture important features of micro-scale malaria transmission, especially in low transmission settings. As these types of settings will become more and more common as malaria infection rates decline, it will be very beneficial to incorporate spatial approaches into prediction frameworks aimed at informing malaria control in low transmission and pre-elimination settings.

## Supporting Information

S1 Table Results from Literature Review on *msp2* MOI *vs*. Malaria Prevalence. The search query ‘falciparum multiplicity infection prevalence msp2’ resulted in 33 hits. Data for all ages was used.
(XLSX)

S1 Text Further Information Regarding the Relationship of MOI *vs*. Age and Heterogeneity of Mosquito Population in Low *vs*. High Transmission Settings. (DOC)

S1 Video Graphical Illustration of our Assumptions Regarding the Expansion of the Mosquito Population. The video shows a gradual increase of mosquito numbers based on our assumptions. At low transmission (low mosquito numbers), mosquitoes are clustered around specific sites inside the modelling area. As transmission increases, the mosquito populations in these sites expand. Further increase of mosquito numbers is assumed to occur homogeneously across the entire modelling area.
(MP4)
