# Supplementary material for: Spatial Effects on the Multiplicity of Plasmodium falciparum Infections
Source: PLoS One. 2016 Oct 6;11(10):e0164054. doi: 10.1371/journal.pone.0164054 (PMC5053403; doi:10.1371/journal.pone.0164054)
Supplement: S1 Text — (DOC) [file pone.0164054.s002.doc]

Supporting Information

Modelling of Spatial Effects on the Multiplicity of *Plasmodium falciparum* Infections

Stephan Karl^1,2*^, Michael White^3^, George Milne^4^, David Gurarie^5^, Simon Hay^6,7^, Alyssa Barry^1,2^, Ingrid Felger^8,9^, Ivo Mueller^1,2,10^

^1^ Population-Based Biology Division, Walter an Eliza Hall Institute of Medical Research, Parkville, Victoria Australia

^2^ Department of Medical Biology, University of Melbourne, Parkville, Victoria, Australia

^3^ MRC Centre for Outbreak Analysis & Modelling, Department of Infectious Disease Epidemiology, Imperial College, London, United Kingdom

^4^ School of Computer Science and Software Engineering, The University of Western Australia, Crawley, WA, Australia

^5^ Department of Mathematics, Applied Mathematics and Statistics, Case Western Reserve University, Cleveland, Ohio, United States

^6^ Department of Zoology, University of Oxford, Oxford, UK

^7^ Fogarty International Center, National Institutes of Health, Bethesda, MD, USA

^8^ Department of Medical Parasitology and Infection Biology Swiss Tropical and Public Health Institute, Basel, Switzerland

^9^ University of Basel, Basel, Switzerland

^10^ Institute of Global Health (ISGlobal), Barcelona

* Corresponding Author

E-mail: karl@wehi.edu.au

# Multiplicity of Infection versus Age

Studies usually show a relationship between the age of human individuals and multiplicity of infection (MOI). This relationship most likely mainly originates from the lower parasite density encountered in older individuals, who have developed clinical immunity to malaria and the related reduced detectability of clones. In the present model, we do not account for the change in parasite density that goes along with acquired immunity and age. Therefore, we do not expect to reproduce the age dependency of MOI observed in field studies. If we do account for reduced detectability of clones with increasing age (still assuming an even distribution of clones in the blood), the (otherwise unchanged) model can reproduce the basic pattern of the observed relationship between age and MOI. Assuming detectability decreases approximately linearly with age (as indicated by the work of Felger et al. 2012, Ref.4, Fig. 4 a, Triplet model), maintaining the assumptions made for duration of infection and exposure to mosquito bites and aiming at an overall equilibrium prevalence of ~70% (as observed in the study by Felger et al. 2012, Ref. 4), the model predicts the MOI *vs*. age relationship shown in S1A Figure. The corresponding MOI *vs*. age relationship observed in the field is shown in S1B Figure.


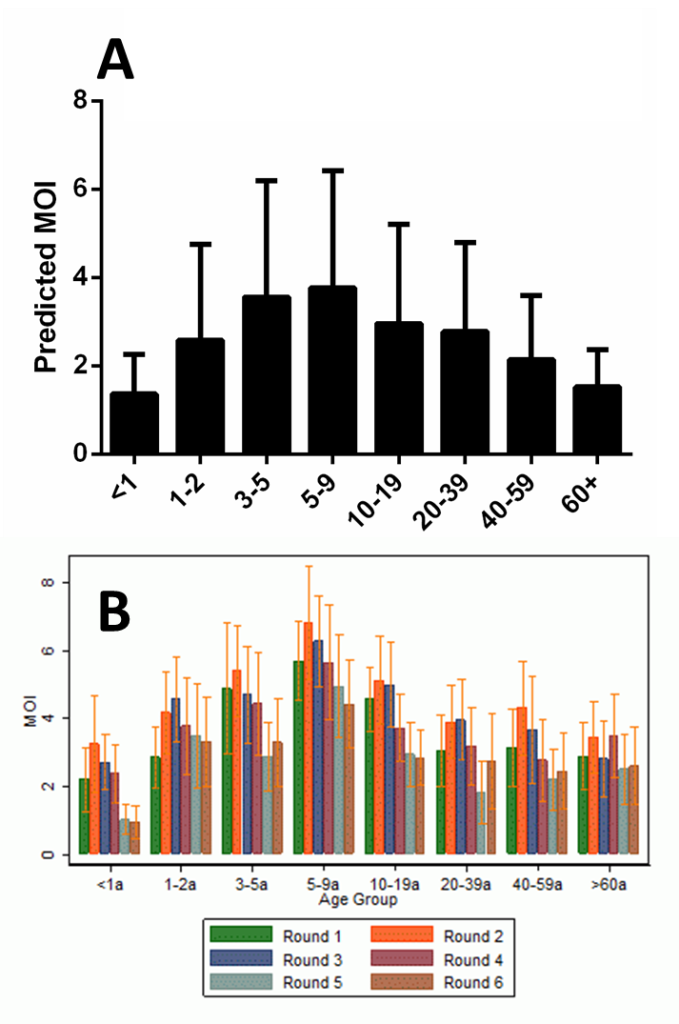


**Figure S1: Comparison of Model Predictions with Field Data for the Relationship between MOI and Age.** Panel A shows MOI versus age model predictions when assuming a linear decrease in detectability of clones with age. Panel B shows field observations of Felger et al.

# Quantification of heterogeneity of transmission in high versus low transmission settings

In order to further substantiate the hypothesis that transmission is more heterogeneous in low transmission settings, we have reanalyzed data by Keating *et al.* reporting mosquito numbers per household in Kenyan villages. S2 Figure shows that the coefficient of variation in mosquito numbers per household, decreases with increasing mean catch numbers, indicating that, indeed, mosquitoes are more homogenously distributed in high transmission settings, which is an important assumption in the present model.

**
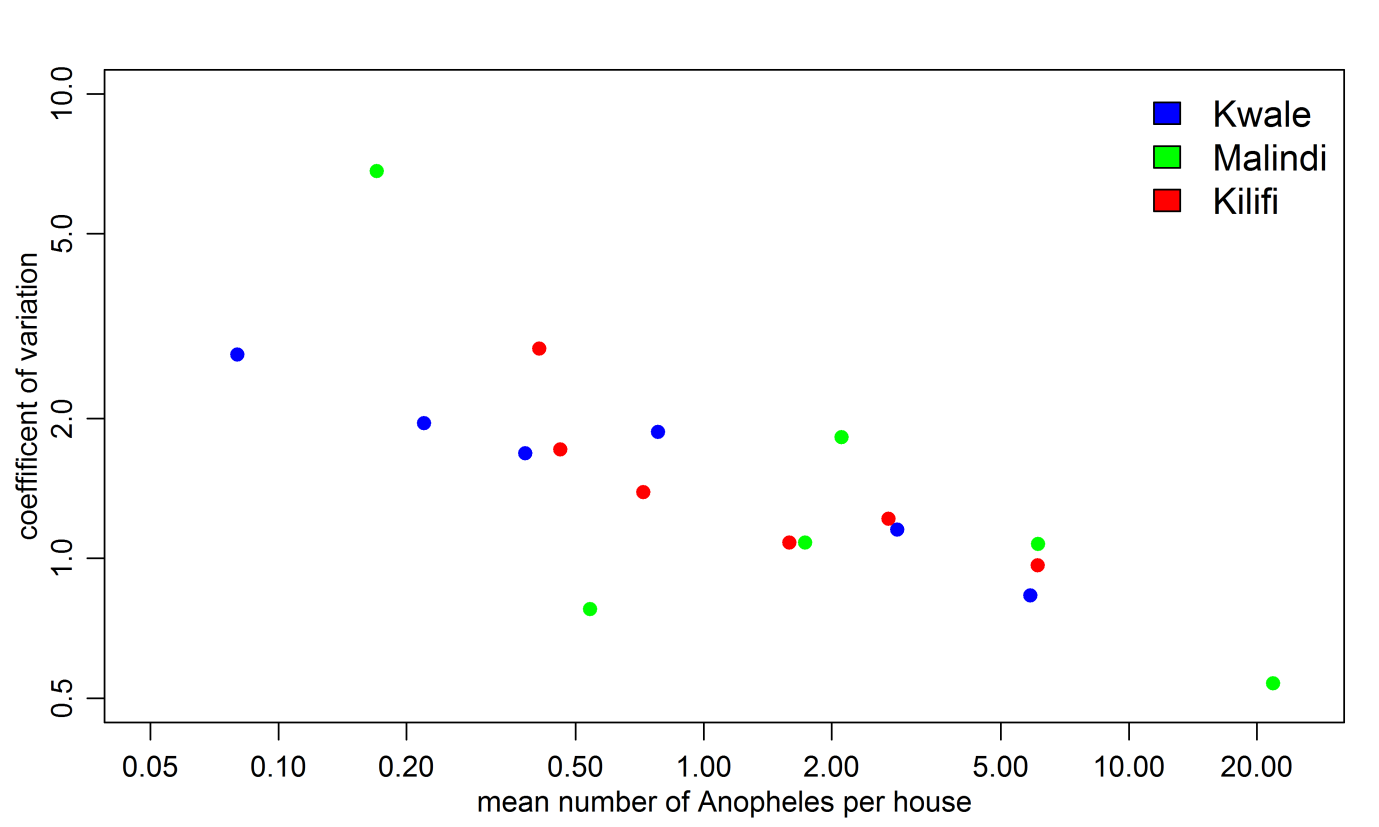
**

**Figure S2: Coefficient of Mosquito Number Variation per Household, with Changing Overall Mosquito Number.**

# Supporting Information References

1. Barry AE, Schultz L, Senn N, Nale J, Kiniboro B, Siba PM, et al. (2013) High levels of genetic diversity of Plasmodium falciparum populations in Papua New Guinea despite variable infection prevalence. Am J Trop Med Hyg 88: 718-725.

2. Fraser-Hurt N, Felger I, Edoh D, Steiger S, Mashaka M, Masanja H, et al. (1999) Effect of insecticide-treated bed nets on haemoglobin values, prevalence and multiplicity of infection with Plasmodium falciparum in a randomized controlled trial in Tanzania. Trans R Soc Trop Med Hyg 93 Suppl 1: 47-51.

3. Smith T, Beck HP, Kitua A, Mwankusye S, Felger I, Fraser-Hurt N, et al. (1999) Age dependence of the multiplicity of Plasmodium falciparum infections and of other malariological indices in an area of high endemicity. Trans R Soc Trop Med Hyg 93 Suppl 1: 15-20.

4. Felger I, Maire M, Bretscher MT, Falk N, Tiaden A, Sama W, et al. (2012) The dynamics of natural Plasmodium falciparum infections. PLoS One 7: e45542.

5. Keating J, Mbogo CM, Mwangangi J, Nzovu JG, Gu V, Regens JL, et al. (2005) Anopheles gambiae s.l. and Anopheles funestus mosquito distributions at 30 villages along the Kenyan coast. J Med Entomol 42: 241-246.
